# DETELPROG Study. Effectiveness of a New Model of Scheduled Telephone Referral from Primary Care to Internal Medicine. A Randomised Controlled Study

**DOI:** 10.3390/jcm8050688

**Published:** 2019-05-16

**Authors:** Luis Miguel Azogil-López, Juan José Pérez-Lázaro, Esther María Medrano-Sánchez, Juan Gómez-Salgado, Valle Coronado-Vázquez

**Affiliations:** 1 Family and Community Medicine, Valverde del Camino Health Centre, 21600 Huelva, Spain; luismiazogil@gmail.com; 2 Andalusian School of Public Health, 18080 Granada, Spain; juanjose.perez.easp@juntadeandalucia.es; 3 Biosanitary Research Institute of Granada, 18080 Granada, Spain; 4 School of Nursing, Physiotherapy and Podiatry, University of Sevilla, 41013 Sevilla, Spain; emedrano@us.es; 5 Department of Sociology, Social Work and Public Health, University of Huelva, 21007 Huelva, Spain; 6 Safety and Health Posgrade Program, Universidad Espíritu Santo, Guayaquil 091650, Ecuador; 7 General Directorate of Health Assistance-Aragonese Health Service, 50071 Zaragoza, Spain; mvcoronado@msn.com

**Keywords:** referral, eConsult, curbside consultation, telemedicine, primary health care, waiting list, patient safety, quality of health care

## Abstract

In Spain, the average waiting time for a specialist consultation is 58 days. A determinant factor that contributes to this situation is the poor communication between primary care and specialised care, which is mainly due to the waiting days for a consultation, number of avoided/avoidable face-to-face referrals, and waiting days for the resolution of the process. DETELPROG is a referral system in which the family physician requests a scheduled outpatient internal medicine consultation, integrated into the usual consultations agenda of both physicians, the family, and the outpatient clinic physician, in order to have a telephone consultation. A randomized controlled clinical trial has been carried out to assess the effectiveness of DELTELPROG. In a sample of 255 patients, the experimental group was referred via a scheduled telephone call, and those in the control group, by face-to-face hospital consultation area. The results showed statistically significant differences between both groups of 27 days (95% confidence interval (CI): 20–33) regarding specialised consultation, 47 days (95% CI: 17–74) as for the resolution of the process, and 91.7% for avoided face-to-face consultations. The DETELPROG resulted as a low coverage system (53%), which makes it a complementary referral model. It is necessary to make an in-depth analysis of the causes that have led to this technologically low coverage.

## 1. Introduction

In Spain, as of 30 June 2017, the average waiting time for a specialist consultation is of 58 days [1], and the perception of patients about the evolution of these waiting lists is negative: 46.3% of patients think that it remains the same as the previous year and 28% of patients believe that it has worsened. Less than 9% of patients think that it has improved, according to a recent Health Department survey [2].

This problem is also present in the vast majority of developed countries [3,4,5], as Canada, as resulted in the *Commonwealth Fund Survey* [5], or United Kingdom, Norway, Finland, the Netherlands, Denmark, Scotland, New Zealand, Canada, Australia, and the United States of America (USA), where various programmes aimed at solving one of the major favouring factors are being implemented: poor communication between primary care and specialised care [4,5].

There are several ways [6,7,8] to improve such communication, but it is telemedicine, including asynchronous consultations platforms [3,4,5,9,10,11,12,13,14,15] (e-Consultations) and real-time telephone consultations (Curbside Consultation) [16,17,18,19,20,21,22], which has opened new possibilities to health care, obtained particularly good results in the waiting times for the first consultation, decreased face-to-face specialised consultations, and increased patients and professionals’ satisfaction [3,5,11,12,16,17,23]. However, studies that provide data on the waiting times until the resolution of the problem that gave rise to the referral have not been found. In addition, e-Consultations require important investments and institutional involvement, as well as leadership and working hours on the part of physicians [12]. With real-time telephone consultations, communication problems [15] were detected of incomplete or fragmented information [7,9,10,11,12,13,14,15,19,20,21,22,23], difficulties to choose the professional to be consulted [24], unpredictable interruptions that are time consuming, as they are not scheduled, etc. [18].

In Spain, health care is a right of citizens and public funds provide its funding. However, the organisation itself is subrogated to the regional governments, which territorially divide their surface in health areas to manage the provision of health care. Each of these health areas consists of several basic areas of primary care and a hospital, where patients from each area could be referred to, if necessary. Each basic primary care area includes one or more primary care medical centers, with one or more family physicians in each. Each patient is assigned a family physician in relation to proximity to his/her home. The patients can change their family physician whenever they wish, that is, they can choose their family physician among the ones working at the closest medical center to their home.

Our health area, the North of Huelva Health Management Area (NHHMA), depends on the regional government of Andalusia, in southern Spain. It is a rural area, which consists of six basic primary care areas and a district hospital of second level, where face-to-face referral is the only possibility for FPs (family physician) to provide a specialised assessment, there is a chronic saturation in the outpatient internal medicine consultations (OIMC), since these assume their own referrals, but also those of several overloaded specialties (cardiology, pulmonology, gastroenterology...), and of other specialties that are not available at our hospital (rheumatology, neurology, nephrology...). Faced with this situation, it was decided to carry out this study, in which a system of scheduled telephone referral (DETELPROG, for its acronym in Spanish) was created as an alternative to face-to-face referral, in which the FP requests a scheduled OIMC, which is integrated into the usual consultations agenda of both physicians, in order to have a telephone consultation with the internist while the patient is in the FP consultation. The objective is to improve the waiting times regarding face-to-face referral and to avoid unnecessary face-to-face consultations, eliminating the barriers of other types of telemedicine described in the previous section.

In the first phase of the study [25], a reduction in waiting times for the first internal medicine consultation (IMC) was found. Now, an assessment of the existence of differences in the waiting times for the resolution of the process between the groups and the number of face-to-face consultations that were avoided in the experimental group will be carried out.

## 2. Materials and Methods

### 2.1. Design

Randomised controlled clinical trial of two arms (experimental and control) without blinding. Blinding was not possible due to the impossibility to blind a face-to-face referral where the patient physically goes to the hospital as compared to the DETELPROG, in which the patient is at the family physician’s office.

### 2.2. Sample

Patients who lived in one of the six basic health areas of the North of Huelva Health Management Area (NHHMA) required a referral to OIMC.

### 2.3. Sample Size

The fisterra.com [26] sample calculator was used for the comparison of the two samples in order to obtain a representative sample, estimating the inclusion of 85 patients per group (experimental and control) and considering a 15% expected proportion of dropouts.

### 2.4. Randomisation

A randomisation of the FPs with quota was performed in the NHHMA (randomisation of patients by blocks), and they were ordered by decreasing number of referrals to IM (Internal medicine) in 2014. Subsequently, they were informed and formed by a one-hour training meeting on the project to each of the FP. Those FP who agreed to participate and signed an informed consent were randomly divided into experimental group and control group using a table of random numbers [27]. The randomization procedure was performed with the FP. FP were randomly assigned either to the experimental or the control group. All of the patients that were associated to each FP were assigned to the same group than their FP. The FPs, already sorted in both groups, assessed for eligibility to every patient who required a referral, according to the inclusion and exclusion criteria. Subsequently, eligible patients were informed, and written consent was obtained. In this way, those in the control group referred patients via face-to-face and only collected the required data from their patients, and those from the experimental group referred patients via DETELPROG. As an exclusion criteria for the FP, those FPs, who, belonging to the control group, did not collect data on at least 50% of their referred patients to IM were excluded, as well as those belonging to the experimental group who did not request any DETELPROG.

### 2.5. Intervention

To carry out the DETELPROG, four appointments of 15 min each were included in the IM consultations agendas to replace two of the appointments of 30 min that are usually scheduled for each patient who attends the corresponding physician’s office for the first time. These appointments were set up by FPs and, subsequently, they set up, in their own consulting room, the same day and at the same time, three consultations followed by six minutes, so that both physicians could have time for the telephone conversation. The referral periods were from 1 March to 10 June and from 3 October to 12 December 2016, when each FP referred their patients through DETELPROG or in-person consultation, according to their group. On the day of the appointment, in the experimental group, the FP called an IMC phone number that was specifically designed for this purpose, with the patient present and the hands-free option of the connected telephone. In this consultation, the treatments, complementary tests, and predictable reviews were agreed via telephone or in person, both professionals leaving record of everything in the patient’s digital record. The requests, consents, and corresponding appointments for complementary tests were sent to the patient’s address, and these would have to be performed either at the health centre (blood tests, X-ray tests) or at the hospital. All of the patients were subsequently followed up for a year or up to discharge from IMC.

### 2.6. Inclusion Criteria

Patients who required a referral to IMC at the discretion of their FP.

### 2.7. Exclusion Criteria

- Patients whose FP, belonging to the experimental group, considered that they would not benefit from the telephone referral for any reason and requested a face-to-face referral.
- Patients who did not agree to telephone referral, even if their FP belonged to the experimental group.

### 2.8. Dropouts Criteria

- Patients who did not attend the requested complementary tests.
- Patients who did not attend the requested reviews.
- Patients who died during the study.
- Patients whose process had not been resolved within the year of follow-up.

### 2.9. Registered Variables, Measurement Techniques and Follow-up of Participants

As independent variables of the patients, the following were recorded: intervention, sex, age, degree of rurality of the population in which live [28] (index indicating the degree of rurality, being zero the average value for rurality. For its elaboration, the ageing of the population, economic dependence, agricultural occupation, habitability of the dwellings, and population density were taken into account), and the distance to the Hospital of Riotinto from their population.

As result variables, the following were assessed:

- Waiting days for IM consultation as the number of days elapsed between the day of the consultation request by the FP and the day of the same.
- Number of avoided/avoidable face-to-face referrals, such as those patients who have had no medical review in IMC or who had had just one, since the latest medical review at IMC is always performed as a non-face-to-face consultation for the completion of the discharge report that will be sent by post. Therefore, in patients with 0 or 1 reviews belonging to the experimental group, the face-to-face consultation will be avoided, and, in the control group, the face-to-face consultation could be avoidable.
- Waiting days for the resolution of the process as number of days elapsed from the day of referral to IMC until the day of resolution of the process (maximum one year), when considering this day as the one in which the FP records in the digital medical history that the problem for which the patient was referred has been resolved, or the day in which this physician draws up the discharge report.

**Figure 1:**
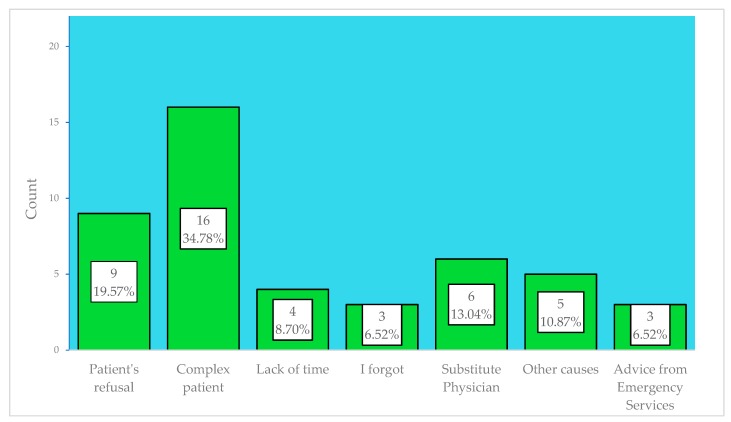
Causes of exclusion to telephone referral.

Patients’ cases of exclusion were also collected. For this, a questionnaire was created for the FPs, where the following causes appeared: patient’s refusal, and six possible causes of FPs’ refusal (Figure 1), based on the most frequent barriers that were detected in other studies [15,18,19,24] and on some problems detected in this study.

The variables record was carried out in two ways: through a data collection sheet and through the already existing data in the digital clinical history system of the Andalusian health system.

The follow-up of each patient was carried out for one year or until the day of the resolution of the process.

### 2.10. Statistical Analysis

The following independent variables regarding the patients were assessed: patients in the experimental and control groups, patients who were excluded, and patients removed after estimations of sex frequencies, as well as the median calculation (25 percentile–75 percentile) in the other three variables, for failing to meet normality criteria.

To assess the existence of statistically significant differences between groups regarding the independent variables, the chi squared test was calculated for sex, and the non-parametric test by Kruskall–Wallis for the other three variables, with a level of significance of 0.05 and a confidence interval of 95%.

Calculating the frequencies for avoided/avoidable consultations, and median (25 percentile–75 percentile) for the other two variables, for not meeting the normality criteria either, were uses for descriptively assessing the outcome variables. Subsequently, whether there were statistically significant inter-group differences was analysed using the chi squared technique for the first variable and, for the other two, through the Mann–Whitney U non-parametric test, with a significance level of 0.05 and a confidence interval of 95%, subsequently applying the Hodges–Lehman median difference estimator for independent samples.

A descriptive analysis of the causes of withdrawal was also conducted by calculating frequencies, thus analysing the existence of statistically significant differences between the experimental and the control groups through the chi squared technique.

### 2.11. Ethical and Legal Aspects

The Research Ethics Committee of the Province of Huelva has evaluated and authorized this study. In addition, as a randomised controlled trial, it has been registered with Trial Registration Number ACTRN12617001536358.

## 3. Results

The 17 FPs of the control group referred 164 patients, of whom 63 (38.4%) were withdrawn from the study. Table 1 describes the sociodemographic characteristics of the population.

**Figure 2:**
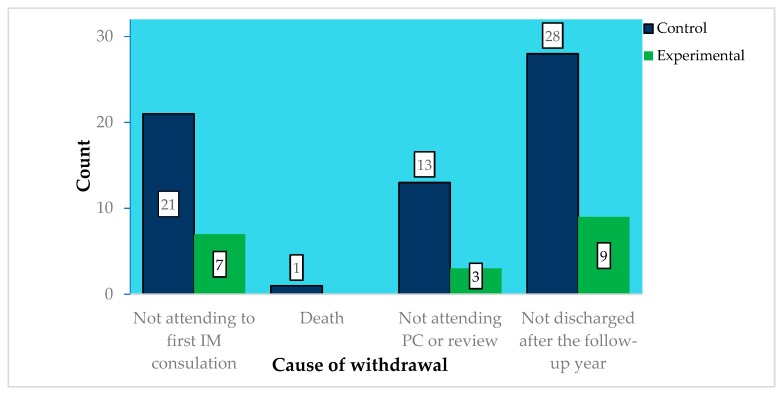
Withdrawn patients’ count by withdrawal cause.

The 13 FPs of the experimental group referred 172 patients, of which 91 (53%) were referred through DETELPROG and 81 (47%) through face-to-face consultation. These last 81 patients were excluded for meeting one of the exclusion criteria, being Primary care (PC) difficulty for patients’ management the most common criterion (Figure 1). Of the 91 patients in the experimental group, 19 (20.9%) were withdrawn from the study for meeting one of the withdrawal criteria (Figure 2), with there being statistically significant differences regarding the number of withdrawn patients between the experimental and the control groups (38.4% vs. 20.9%; *p* = 0.04), but not in the distribution of withdrawals according to their causes *p* = 0.88.

Table 2 describes the patients’ independent variables, finding statistically significant differences regarding the distance to the hospital and the degree of rurality of the area.

Regarding the patients’ diseases, Table 3 summarizes the results of the medical specialty, where the patients were referred.

With regard to the result variables (Table 4), 91.7% of the DETELPROG avoided a face-to-face consultation with the internist, with 85.1% of the control group consultations being predictably avoidable and not finding statistically significant differences between both groups.

Regarding the waiting days for IM consultation and up to the resolution of the process, statistically significant differences were found between both of the groups (*p* ˂ 0.001 and *p* = 0.002 respectively), with a difference of 27 (CI 95%: 20–33) and 46 (CI: 95%: 17–74) days, respectively.

During the study, 9.3% of the total time designed for first consultations in PC was devoted to DETELPROG. DETELPROG represented about 9% of all the referrals to IM. Thus, this process avoided the other two types of referral.

## 4. Discussion

In this study, we found that DETELPROG, as compared to face-to-face referral (the only existing model for referral in the NHHMA), considerably decreases the waiting days for the resolution the process for which the patient is referred, avoiding face-to-face specialised consultation in almost all cases.

In the Canadian RACE program [22], one of the most powerful immediate telephone consultation programs in the world, there is no waiting any longer, as the call is answered in real time. However, only 60% of the telephone consultations avoids face-to-face consultation, in contrast to the 91% avoided with DETELPROG.

With regard to e-consultations, the waiting days for the specialist’s response are between 0 and four days, but data on avoided face-to-face specialist consultations are highly variable, between 12% and 85% [3,4,5,14].

In the case of regular meetings with a reference specialist, in an Andalusian study also on PC and IM [7], only 73% of face-to-face consultations were avoided.

Data on the waiting days for the resolution of the process have not been found in the studies, which was considered to be the most important measure in this study for being the actual time elapsed until the resolution of the patient’s problem, and because this is considerably reduced with DETELPROG when compared to face-to-face referrals.

As for the differences that were found between the groups regarding the distance to the hospital, the most relevant difference is found between the experimental and the control group in 10.4 km (19.6–5.1). Thus, it is possible to suggest that patients who live closer to the hospital are more likely to reject the telephone referral, although the differences in distance are so small that it is not possible to confirm this idea, based on the data that were obtained in this study. As for the degree of rurality, a possible tendency is detected to not using new technologies for referrals, although the differences, albeit minimal, go in the opposite direction.

As for the low coverage of DETELPROG (53% of the patients in the experimental group), we believe it to be necessary to undertake a qualitative study to analyse barriers, difficulties, and possible areas of improvement regarding DETELPROG at greater length, so as to optimise this type of referral and make it possible to implement this system in our health area, and even export it to other areas.

Therefore, we consider that DETELPROG, as compared to face-to-face referral (the only existing model for referral in the NHHMA), considerably decreases the waiting days for the resolution the process for which the patient is referred (parameter not valued in studies on other models of referral), avoiding face-to-face specialised consultation in almost all cases.

The reduction of waiting days for the first IM consultation with DETELPROG is not as relevant as it is in the case of real-time telephone consultations or in the case of referrals through electronic platforms. However, it is a referral method that does not require any extraordinary economic or political support, which reduces waiting days with respect to face-to-face referral, and that does not imply the barriers of real-time telephone consultations that are caused by immediacy and a lack of programmability. In addition, we cannot forget that this is the model that more face-to-face referrals avoid according to the data that are found in the literature.

We also believe that this model of scheduled telephone referral would be applicable to other specialties in which referrals with good results were already applied through asynchronous electronic platforms and/or immediate telephone consultations [14,22], both in rural and urban environments. However, specific studies would be needed in these specialties and other areas to support this hypothesis.

As a limitation, we consider that the reduction of waiting days is lower than in the other models of real-time telephone consultations and e-consultations, but no additional expenditure or involvement of the administrations is required for DETELPROG, as is the case of e-consultations, being this a model that is only applicable if professionals request it. On the other hand, with this new type of referral, no problems are derived from the immediacy and the lack of programmability of real-time telephone referrals.

## 5. Conclusions

As in the previous article, we do not consider DETELPROG for complex patients (34.8% of patients excluded) [25], whose diagnoses are not more or less outlined from PC or regarding patients who require a more specialised clinical management or examination. However, we believe that the low coverage of DETELPROG (53%) does not make it an exclusive referral model, but a complement to other techniques. It necessary to conduct in-depth analysis of the causes that have led to this technologically low coverage.

## Figures and Tables

**Table 1:** Sociodemographic characteristics of the included and excluded family physicians (FPs) by groups.

Sociodemographic Characteristics	FPs Excluded (*n* = 20)	Experimental Group (*n* = 13)	Control Group (*n* = 17)	*p*-Value
Sex *n* (%)				
Male Female	13 (65%) 7 (35%)	7 (53.8%) 6 (46.8%)	14 (82.4%) 3 (17.6%)	0.236
Age in years ^a^	57.5 (36–60)	59 (43–61.5)	54 (43–58.5)	0.624
Distance to hospital in kilometres ^b^	44.5 (± 25.4)	3.9 (± 11.4)	32 (± 24.4)	0.551
Health insurance cards adjusted by age ^b^	1.747 (± 590)	1.756 (± 635)	1.804 (± 570)	0.926
N of referrals in 2014 ^a^	32 (22–45)	27 (20–49)	21 (18–32)	0.101
Rurality degree ^b^	−0.53 (± 0.54)	−0.57 (± 0.41)	−0.57 (± 0.51)	0.768

a: Median (quartile range P25–P75); b: Mean (± standard deviation). FP: family physician.

**Table 2:** Sociodemographic characteristics of the included and excluded patients by groups.

Sociodemographic Characteristics	Excluded Patients (*n* = 81)	Withdrawn Patients (*n* = 82)	Experimental Group (*n* = 72)	Control Group (*n* = 101)	*p*-Value
Sex *n* (%)					
Male Female	33 (40.7%) 48 (59.3%)	33 (40.2%) 49 (59.8%)	31 (43.1%) 41 (56.9%)	41 (40.6%) 60 (59.4%)	0.985
Age in years ^a^	56(44.42–69.50)	54.8(39.75–71.34)	56 (41.25–66)	56 (43–69.30)	0.92
Rurality degree ^a^	−0.9(−0.92–−0.59)	−0.78(−1.09–−0.59)	−0.69(−0.92–−0.42)	−0.78(−1.13–−0.43)	0.02
Distance to hospital in kilometres ^a^	30.5 (27–35)	27 (3.7–41.22)	36.2 (27–44.9)	23 (0–41.3)	˂0.01

a: Median (quartile range P25–P75).

**Table 3:** Frequency of referral diseases by groups.

Medical Specialty		Group		Total
		Control (*n*)	Experimental (*n*)	
Referral disease	Cardiovascular	15	11	26
	Endocrinology	6	5	11
	Neurology	9	2	11
	Gastroenterology	52	33	85
	Oncology	0	2	2
	Nephrology	1	2	3
	Rheumatology	4	4	8
	Others	14	13	27
Total		101	72	173

**Table 4:** Summary and differences between groups of the dependent variables.

Variable	Experimental Group (*n* = 72)	Control Group (*n* = 101)	*p*-Value	Medians Differences in Days (CI 95%)
Avoided/avoidable face-to-face referrals *n* (%)	66 (91.7)	86 (85.1)	*p* = 0.290	Not statistically significant differences
Waiting time for IM consultation, in days ^a^	11 (7–19)	41 (23–56)	*p* < 0.001	27 (20–33)
Waiting time for resolution of the process, in days	104.5 (39.5–169)	147 (74–228)	*p* = 0.002	46 (17–74)

a: Median (quartile range P25–P75). IM: internal medicine; CI: confidence interval.
